# Exposure to interparental violence and intimate partner violence among women in Papua New Guinea

**DOI:** 10.1186/s12905-023-02179-5

**Published:** 2023-02-07

**Authors:** Bright Opoku Ahinkorah, Richard Gyan Aboagye, Abdul Cadri, Tarif Salihu, Abdul-Aziz Seidu, Sanni Yaya

**Affiliations:** 1 School of Public Health, Faculty of Health, University of Technology Sydney, Sydney, Australia; 2 Department of Family and Community, Fred N. Binka School of Public Health, University of Health and Allied Sciences, Hohoe, Ghana; 3 Department of Social and Behavioural Science, School of Public Health, University of Ghana, Legon- Accra, Ghana; 4 Department of Family Medicine, Faculty of Medicine, McGill University, Montreal, QC Canada; 5 Department of Population and Health, University of Cape Coast, Cape Coast, Ghana; 6 College of Public Health, Medical and Veterinary Sciences, James Cook University, Townsville, Australia; 7 REMS, Consult, Sekondi-Takoradi, Ghana; 8 Centre For Gender and Advocacy, Takoradi Technical University, P.O.Box 256, Takoradi, Ghana; 9 School of International Development and Global Studies, University of Ottawa, Ottawa, Canada; 10 The George Institute for Global Health, Imperial College London, London, UK

**Keywords:** Demographic and Health Survey, Papua New Guinea, Interparental violence, Intimate partner violence

## Abstract

**Introduction:**

Evidence suggests that childhood exposure to interparental violence increases the risk of intimate partner violence (IPV) experience or perpetration in adolescence or adulthood. However, it is unclear if exposure to interparental violence increases the risk of IPV among women in Papua New Guinea. This study, therefore, seeks to fill this gap in the literature by examining the association between childhood exposure to interparental violence and IPV among women in Papua New Guinea.

**Methods:**

We used data from the most recent 2016–18 Papua New Guinea Demographic and Health Survey. We included 3,512 women in our analyses. Past-year experience of IPV was the outcome variable in this study. Exposure to interparental violence was the key explanatory variable. We used a multilevel binary logistic regression to examine the association between exposure to interparental violence and IPV.

**Results:**

We found a higher probability of experiencing IPV among women exposed to interparental violence [aOR = 1.45, 95% CI = 1.13, 1.86] relative to women who were not exposed. Furthermore, we found that women living in rural areas had a lower likelihood of IPV experience [aOR = O.50, 95% CI = 0.32, 0.80] compared to those in urban settings. Finally, a greater odd of IPV experience was found among women staying in the Highlands Region [aOR = 1.44, 95% CI = 1.06, 1.96] compared to those staying in the Southern Region.

**Conclusion:**

Exposure to interparental violence was found to be significantly associated with IPV among women in Papua New Guinea. The findings of this study suggest the need for proven operational strategies to reduce IPV, such as improving anti-IPV laws in Papua New Guinea. We recommend the development and implementation of intercession strategies to reduce the experience and justification of violence among women exposed to interparental violence. In addition, health professionals should implement counseling and health education initiatives to tackle the consequences of IPV on women's well-being.

## Introduction

Intimate partner violence (IPV), a serious public health problem, is defined as physical violence, sexual violence, stalking, or psychological aggression (including coercive acts) by a current or former intimate partner, whether or not the person is a spouse [1, 2]. Experience of IPV is prevalent among individuals across the diverse gender spectrum, including males, females, transgender and nonbinary individuals [3]. Nevertheless, the prevalence of IPV is disproportionately higher among people who self-identify as female compared to people who self-identify as male, with further evidence indicating the likelihood of experiencing IPV to be higher among people who identify as female than people who identify as male [3, 4].

Interparental violence, on the other hand, is violence that occurs between parents [5]. Exposure to interparental violence has mostly been defined as the situation where children see, hear, involve, or experience the aftermath of physical, sexual, or emotional violence that occurs between their caregivers [6]. Exposure to interparental violence during youthful age has detrimental effects on the individual [5, 6]. However, evidence on the prevalence of interparental violence exposure remains scarce in Papua New Guinea (PNG).

The experience of IPV has a huge negative impact on women’s health and well-being. Women who experience IPV report a higher likelihood of medical, gynaecological, and stress-related symptoms compared to women who do not experience IPV [7, 8]. Stress due to the experience of IPV among women has been reported to activate neuroendocrine and immune system pathways, which may increase the risk of chronic conditions, including autoimmune disorders and cancer [9]. Women of reproductive age who experience IPV present with poor reproductive and sexual health, including unintended pregnancy, Human Immunodeficiency Virus (HIV), and other sexually transmitted infections [10–12]. Factors underlying these poor reproductive and sexual health outcomes include forced or coerced sex, a partner’s refusal to use condoms, and other forms of reproductive coercion, such as pressuring a woman to become pregnant against her wishes and sabotaging contraception (breaking or removing condoms during sex) [13]. IPV is also associated with increased risk factors of obstetrical and gynaecologic complications, pregnancy-associated death, preterm birth, low birth weight, peripartum depression, and substance use [14].

IPV has been reported to have an impact on women’s mental health, which translate to healthcare costs and disease burden among women [15]. IPV increases a woman’s likelihood of experiencing depression, post-traumatic stress disorder, anxiety, suicidal behaviour, and substance use behaviour [16–18]. The physical health impact of IPV on women includes injuries such as contusions, lacerations, and fractures [19, 20].

The prevalence of IPV, as well as its associated burden, is highest in most low- and -middle-income countries [21, 22], and PNG is noted to be one of the countries with the highest prevalence, as well as the burden of IPV [23, 24]. The underlying factors that result in the high prevalence of IPV in PNG are the strict gender roles and gender relations in which IPV is used as a means of keeping women in their place and giving men the decision-making power in the relationship [25, 26]. Several other factors are associated with an increased risk of IPV. Some of these factors include substance use, stress, low level of education, ineffective communication in the relationship, unequal power relation, unemployment, gender inequitable masculinities, and harmful attitudes to gender relations that result in female disempowerment and marginalization [27, 28].

Evidence suggests that children's exposure to interparental violence increases their risk of being victims of IPV or perpetrating violence in adolescence or adulthood [29]; however, it is unclear if exposure to interparental violence increases the risk of experiencing IPV among women in PNG. This study, therefore, seeks to fill this gap in literature accordingly, and contribute to efforts toward addressing the high prevalence of IPV in PNG.

In assessing the association between exposure to interparental violence and IPV experience, we used a multilevel logistic regression model, where we considered how individual and community-level factors interact to explain the association between exposure to interparental violence and IPV experience. Given the high prevalence of IPV in PNG [30], we hypothesize that childhood exposure to interparental violence is associated with IPV among women in PNG.

## Methods

### Data source and study design

We used data from the 2016–18 PNG Demographic and Health Survey (PNG DHS). The PNG DHS is a nationally representative survey conducted periodically to provide an update on the demographic and health situation in PNG [31]. The 2016–18 PNG DHS is the first official DHS conducted in PNG in collaboration with the worldwide Demographic and Health Surveys Program, which is a global programme coordinated by Inner City Fund (ICF), based in Rockville, Maryland, USA [31]. The PNG DHS employed a descriptive cross-sectional design in collecting data from the respondents on several indicators such as domestic violence and other related health issues [31]. The dataset used can be accessed at https://dhsprogram.com/data/dataset/Papua-New-Guinea_Standard-DHS_2017.cfm?flag=1. We relied on the Strengthening Reporting of Observational Studies in Epidemiology (STROBE) reporting guidelines in drafting this paper [32].

### Sampling technique and sample size

The PNG DHS employed a two-stage cluster sampling technique in recruiting the respondents for the survey. The sampling method was carried out in two stages. All the provinces were stratified into urban and rural areas and this yielded forty-three sampling strata, except for the National Capital District, which has no rural areas. In the first stage, 800 census units were selected with probability proportional to the census unit size. In the second stage, a fixed number of 24 households per cluster were selected with an equal probability of systematic selection from the newly created household listing, resulting in a total sample size of approximately 19,200 households. Detailed sampling procedure has been highlighted in the literature [31]. In this study, we included 3,512 women with complete observations on all variables of interest.

### Variables

Past-year experience of IPV was the outcome variable in this study. IPV variables were derived from the domestic violence model, which used a modified version of the conflict tactics scale to ask questions [33, 34]. The questions used to assess physical, emotional, and sexual violence have been published elsewhere in the literature [35–39]. The response options to each of the questions were “never” “often” “sometimes” and “yes, but not in the last 12 months”. For this study’s purpose and regarding literature [35–37], we recoded those whose response option was either “never” and “yes, but not in the last 12 months” as “no” and was assigned a value “zero (0)”. The remaining response options “often” and “sometimes” were coded as “yes” and labelled as “1”. We utilised the numeric labels “0 = no” and “1 = yes” in the final analysis.

Exposure to interparental violence was the key explanatory variable in the study. This variable was measured using the question *“As far as you know, did your father ever beat your mother?”*. The response options to this question were “no”, “yes”, and “don’t know”. Those who answered "no" or "don't know" were classified as "Not exposed" to interparental violence, whereas those who answered "Yes" were classified as "Exposed”. The reclassified responses were further coded as “0 = not exposed” and “1 = exposed”. This categorization was informed by literature that utilised the DHS dataset [40, 41].

We included 15 variables as covariates in the study. These variables were selected based on their association with IPV from previous studies [40–45] and their availability in the DHS dataset. The variables consisted of the age of women, educational level, marital status, current working status, exposure to mass media (television, radio, and newspaper or magazine), parity, wealth index, place of residence, region, community socioeconomic status, and community literacy level. We maintained the existing coding for current working status, wealth index, and place of residence in the final analysis as found in the DHS. Age was recoded into “15–24”, “25–34″, and “35 and above”. Educational level was recoded into “no education”, “primary”, and “secondary or higher”. Exposure to mass media was coded into “none”, “one”, and “two or more”. Parity was coded as “0 birth”, “1 birth”, “2 births”, “3 births”, and “4 or more births”. Both community socioeconomic status and literacy level were coded as “low”, “medium”, and high”.

### Statistical analyses

**Fig. 1:**
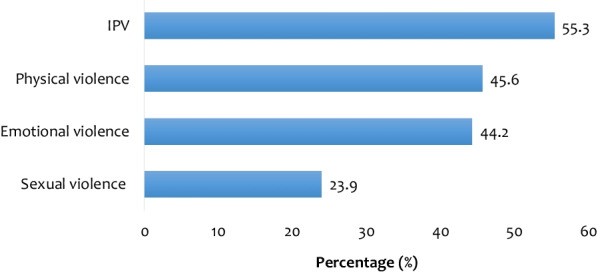
Prevalence of IPV among women in Papua New Guinea

We used Stata software version 16.0 (Stata Corporation, College Station, TX, USA) throughout the analysis. First, we employed percentages to summarise the results of the prevalence of IPV (Fig. 1). Pearson chi-square test was later used to examine the relationship between the explanatory variables and IPV (Table 1). Subsequently, we used a multilevel binary logistic regression to examine the association between exposure to interparental violence and IPV, controlling for the covariates (Table 2). Five models were built to examine the association between interparental violence and IPV. Before the regression analysis, a multicollinearity test was conducted using the variance inflation factor (VIF). The results showed that the minimum, maximum, and mean VIFs were 1.02, 3.95, and 2.08, respectively. Hence, there was no evidence of multicollinearity among the variables studied. The first model (Model O), which was an empty model with no explanatory factors or covariates, indicated the variation in IPV ascribed to the primary sampling units (PSUs). Model I contained only the key explanatory variable, whereas Model II included the key explanatory variable and individual-level covariates. Model III contained the key explanatory variable and the community level variables. The final model (Model IV) consisted of the key explanatory variable and all the covariates. The results of the regression analysis were presented in a tabular form using crude odds ratio (cOR) and adjusted odds ratios (aOR) with 95 percent confidence intervals (CIs). Statistical significance was set at *p*< 0.05. Furthermore, each of the five models incorporated both fixed and random effects. Fixed effects represented the association between the exposure to interparental violence and/or covariates and IPV, whereas random-effects denoted the measure of variation in the IPV dependent on primary sampling units measured by intra-cluster correlation coefficient (ICC) . The Akaike's Information Criterion (AIC) was used to measure model fitness. The multilevel regression models were run using Stata's "melogit" function. The "svyset" command was used to correct for disproportionate sampling and non-response, and weighting was done to account for the complex nature of DHS data. All the analyses were weighted according to the DHS guidelines.

**Table 1:** Distribution of intimate partner violence across exposure to interparental violence and the covariates

Variable	Weighted		Intimate partner violence	
	Frequency	Percentage	Yes [% CI]	*P* value
*Exposed to interparental violence*				< 0.001*
No	1874	53.4	49.4 [45.8–53.0]	
Yes	1638	46.6	62.0 [57.1–66.7]	
*Women’s age (Years) [mean *= *32.5, standard deviation* = *7.92)*				< 0.001*
15–24	769	21.9	61.9 [56.5–67.0]	
25–34	1329	37.8	58.7 [53.4–63.5]	
35–49	1414	40.3	48.5 [44.0–53.1]	
*Women’s educational level*				0.163
No education	1001	28.5	49.4 [42.8–56.0]	
Primary	1637	46.6	55.3 [51.6–58.9]	
Secondary or higher	874	24.9	62.1 [50.0–72.8]	
*Marital status*				0.902
Married	2902	82.6	55.4 [51.5–59.1]	
Cohabiting	610	17.4	55.0 [49.3–60.5]	
*Current working status*				0.268
No	2395	68.2	54.3 [49.7–58.7]	
Yes	1117	31.8	57.5 [53.4–61.5]	
*Parity*				0.095
Zero birth	339	9.7	60.0 [51.4–68.0]	
1 birth	633	18.0	65.9 [52.5–77.2]	
2 births	563	16.0	52.6 [43.9–61.2]	
3 births	576	16.4	55.2 [49.7–60.5]	
4 or more births	1401	39.9	50.5 [46.0–55.0]	
*Exposure to mass media*				< 0.001*
None	1853	52.7	49.4 [45.7–53.2]	
One	607	17.3	57.4 [51.3–63.3]	
Two or more	1052	30.0	64.4 [59.1–69.4]	
*Wealth index*				0.055
Poorest	659	18.8	50.8 [44.3–57.3]	
Poorer	695	19.8	50.6 [44.7–56.5]	
Middle	703	20.0	54.9 [49.0–60.6]	
Richer	663	18.9	56.8 [51.8–61.8]	
Richest	792	22.5	62.2 [54.3–69.5]	
*Place of residence*				0.005*
Urban	395	11.2	64.8 [58.3–70.8]	
Rural	3117	88.8	54.1 [50.3–57.9]	
*Region*				0.444
Southern Region	682	19.4	52.1 [47.5–56.6]	
Highlands Region	1347	38.4	56.0 [51.1–60.8]	
Momase Region	996	28.3	58.1 [49.5–66.2]	
Islands Region	487	13.9	52.2 [47.1–57.2]	
*Community literacy level*				0.076
Low	1362	38.8	50.6 [45.6–55.6]	
Medium	1271	36.2	57.2 [52.5–61.8]	
High	879	25.0	59.8 [52.1–67.0]	
*Community socioeconomic status*				0.079
Low	1981	56.4	51.6 [48.0–55.1]	
Medium	307	8.7	62.8 [48.3–75.3]	
High	1224	34.9	59.5 [53.2–65.4]	

*P value were generated from the Chi-square test

**Table 2:** Association between exposure to interparental violence and intimate partner violence among women in Papua New Guinea

Variable	Model O	Model I cOR [95% CI]	Model II aOR [95% CI]	Model III aOR [95% CI]	Model IV aOR [95% CI]
Fixed effects results					
*Exposed to interparental violence*					
No		1.00	1.00	1.00	1.00
Yes		1.53** [1.17, 2.00]	1.46** [1.13, 1.87]	1.52** [1.16, 1.98]	1.45** [1.13, 1.86]
*Women’s age (years)*					
15–24			1.00		1.00
22–34			1.12 [0.67, 1.88]		1.14 [0.68, 1.89]
35–49			0.69 [0.39, 1.24]		0.69 [0.39, 1.23]
*Women’s educational level*					
No education			1.00		1.00
Primary			1.28 [0.79, 2.09]		1.32 [0.79, 2.22]
Secondary or higher			1.25 [0.48, 3.23]		1.29 [0.47, 3.53]
*Marital status*					
Married			1.00		1.00
Cohabiting			1.06 [0.81, 1.41]		1.05 [0.79, 1.40]
*Current working status*					
No			1.00		1.00
Yes			1.17 [0.91, 1.49]		1.18 [0.92, 1.51]
*Parity*					
Zero birth			1.00		1.00
1 birth			1.01 [0.61, 1.68]		1.02 [0.62, 1.71]
2 births			0.60 [0.30, 1.21]		0.61 [0.31, 1.23]
3 births			0.73 [0.43, 1.24]		0.74 [0.44, 1.25]
4 or more births			0.77 [0.49, 1.21]		0.80 [0.51, 1.25]
*Exposure to mass media*					
None			1.00		1.00
One			1.12 [0.80, 1.57]		1.07 [0.77, 1.50]
Two or more			1.38 [0.93, 2.05]		1.28 [0.87, 1.88]
*Wealth index*					
Poorest			1.00		1.00
Poorer			0.97 [0.67, 1.40]		0.95 [0.66, 1.38]
Middle			1.09 [0.74, 1.61]		1.06 [0.72, 1.56]
Richer			1.01 [0.67, 1.54]		0.90 [0.57, 1.41]
Richest			0.94 [0.55, 1.60]		0.68 [0.37, 1.25]
*Place of residence*					
Urban				1.00	1.00
Rural				0.56** [0.40, 0.78]	0.50** [0.32, 0.80]
*Region*					
Southern Region				1.00	1.00
Highlands Region				1.37* [1.02, 1.83]	1.44* [1.06, 1.96]
Momase Region				1.15 [0.85, 1.56]	1.18 [0.86, 1.61]
Islands Region				1.03 [0.76, 1.38]	1.03 [0.75, 1.40]
*Community literacy level*					
Low				1.00	1.00
Medium				1.22 [0.93, 1.60]	1.10 [0.80, 1.51]
High				1.14 [0.82, 1.61]	1.02 [0.67, 1.56]
*Community socioeconomic status*					
Low				1.00	1.00
Medium				1.38 [0.86, 2.19]	1.45 [0.90, 2.35]
High				1.28 [1.00, 1.63]	1.35 [0.99, 1.86]
*Random effects results*					
PSU variance (95% CI)	0.830 [0.609–1.130]	0.806 [0.590–1.103]	0.795 [0.558–1.132]	0.752 [0.546, 1.035]	0.777 [0.550–1.098]
ICC	0.201	0.197	0.194	0.186	0.191
Wald chi-square	Reference	9.64 (0.002)	39.27 (0.002)	35.90 (< 0.001}	57.42 (< 0.001)
*Model fitness*					
Log-likelihood	−2112.8019	−2100.4571	−2063.7284	−2088.4683	−2053.533
BIC	4241.932	4225.406	4282.572	4266.74	4327.492
N	3512	3512	3512	3512	3512
Number of clusters	750	750	750	750	750

*aOR* adjusted odds ratios, *cOR* Crude odds ratio,* CI* Confidence interval, *p< 0.05, **p< 0.01; 1.00 Reference category; *PSU* Primary Sampling Unit, *ICC* Intra-Class Correlation Coefficient, *LR Test* Likelihood ratio Test, *AIC* Akaike’s Information Criterion; *N* Total sample

### Ethical consideration

Because this study used publically available data, no ethical approval was required. Further information regarding the data and ethical norms can be accessed at http://goo.gl/ny8T6X. We carried out this study in accordance with relevant guidelines and regulations concerning the use of DHS dataset for publication.

## Results

### Prevalence of intimate partner violence among women in Papua New Guinea

Figure 1 presents the prevalence of IPV among women in PNG. The overall prevalence of IPV among women was 55.3% [53.6–56.9]. The prevalence of physical, emotional, and sexual violence observed in this study were 45.6, 44.2, and 23.9%, respectively.

### Distribution of intimate partner violence across the explanatory variable and covariates

Table 1 displays the distribution of IPV across the explanatory variable and covariates. The results indicated substantial differences in IPV across the exposure to interparental violence, women’s age, exposure to mass media, and place of residence at *p*< 0.05. Particularly, IPV was found to be prevalent among women exposed to interparental violence [62.0% (57.1–66.7)] relative to women not exposed interparental violence [49.4% (45.8–53.0)]. IPV was highest among women aged 15–19 [61.9% (56.5–67.0)] but lowest among those aged 35–49 [48.5% (44.0–53.1)]. In terms of exposure to mass media, the highest proportion of IPV [64.4% (59.1–69.4)] was observed among women who were exposed to two or more mass media whereas those who had no exposure to mass media recorded the lowest proportion [49.4% (45.7–53.2)]. With the place of residence, IPV was higher among urban women [64.8 (58.3–70.8)] compared to rural women [54.1% (50.3–57.9)]. Finally, apart from exposure to interparental violence, maternal age, exposure to mass media, and place of residence which were found to be significantly associated with IPV in this analysis, the rest of the variables were insignificantly related to IPV (see Table 1).

### Mixed effect analysis of association between exposure to interparental violence and intimate partner violence

#### Fixed effects (measures of association) results

Model III of Table 2 presents the results of the association between interparental violence exposure and IPV among women in PNG, controlling for the covariates. We found a higher probability of experiencing IPV among women exposed to interparental violence [aOR = 1.45, 95% CI = 1.13, 1.86] relative to women who were not exposed. For the covariates, we observed that women who were living in rural settings had a lower likelihood of IPV experience [aOR = O.50, 95% CI = 0.32, 0.80] relative to women living in urban settings. Finally, in terms of region, greater odds of experiencing IPV was found among women staying in the Highlands Region [aOR = 1.44, 95% CI = 1.06, 1.96] relative to those staying in the Southern Region (see Table 2).

#### Random effects (measures of variation) results

As shown in Table 2, in the empty model, there were substantial variations in the likelihood of IPV across the clustering of the primary sampling units (PSUs) (σ2 = 0.83, 95% CI 0.61–1.13). The empty model showed that 20.1% of the total variance in IPV was attributed to the between-cluster variation of characteristics (ICC = 0.201). The between-cluster variations decreased marginally in Model I, from 20.1% in the empty model to 19.7% in the model with only the key explanatory variable (exposure to interparental violence). From Model I, the ICC declined further to 19.4% (ICC = 0.194) in the model that controlled for the individual-level covariates (Model II) and reduced further to 18.6% in the model that controlled for community-level covariates (Model III). In the final model, the ICC value increased to 19.1%. This result shows that the disparities in the probability that IPV would occur could be ascribed to the differences in the grouping of the sampling units. With the lowest log-likelihood ratio (-2053.533) and the highest BIC value (4327.492), the final model which had the key explanatory variable and controlled for both the individual and community level variables was chosen as the best fit for predicting the occurrence of IPV.

## Discussion

The current study examined the association between exposure to interparental violence and IPV among women in PNG. The overall prevalence of IPV was 55.3%. The prevalence found in this study is similar to those of other studies conducted in Asia–Pacific countries including Bangladesh [46] and Afghanistan [47]. However, the finding in this current study is higher than those found in prior studies, which include 39% in India [48], 40% in Pakistan [49], and 45.3% in Bangladesh [50], but lower than 82.7% in rural Bangladesh [51], and 67% in the Gambia [52]. The high prevalence of IPV found in this research may be a result of the country's rigid conventional attitudes and gender standards, as well as poor access to public health education, justice, and social services, which have been reported to influence IPV perpetration [47, 53, 54]. Differences in cultural and socioeconomic dimensions could have had a profound impact on our findings.

Although rates varied depending on the type of abuse, IPV was relatively common in PNG. Generally, physical violence was found in the present study to be the most prevalent form of IPV (45.6%), followed by emotional violence (44.2%), and then sexual violence (23.9%). This tendency was largely consistent with other Asia–Pacific studies on IPV [55]. The extensive history of violent conflicts in PNG, not only affected and included a sizeable section of the current adult population but also left a persistent negative imprint on the inhabitants. This could have influenced the prevalence of physical, emotional, and sexual violence among women, as women usually become the vulnerable populace. Evidence also suggests that men who have experienced childhood trauma were more likely to commit all measurable types of IPV, a plausible reason for the observed findings in our study [55, 56].

Despite the wide cultural diversity in PNG, men are typically socialized to engage in forceful and active interpersonal interaction. In PNG, using violence to settle disputes, express anger, or discipline misbehaving people, especially women who defy social norms, is commonplace and completely justified. Young children are frequently subjected to physical abuse, and the common wisdom holds that physical abuse improves understanding [57]. It is important to note that in PNG, fathers are primarily responsible for enforcing household rules. The PNG home culture normalizes physical abuse of both children and mothers, which is reflected in these strict parenting techniques. That is, strict parenting methods used by men are most significantly related to whether the male spouse physically punishes the children, which is directly tied to male IPV against women in the house, a form of disciplining women [55]. Men who have experienced child abuse or who have seen their mothers being abused are more likely to physically abuse their wives [55].

Furthermore, women who experience sexual violence are seen as acting contrary to social norms that hold women to be obedient to men. Men view women and girls who defy these social conventions and are observed entering pubs or nightclubs as a fair game. Such ideas are based on the idea that violence against women is a means of punitively enforcing male control over women. Acts of sexual violence also have a disciplinary component because men target women who are thought to be acting contrary to social expectations of how women ought to act. Men utilized sexual assault as a tool of control and punishment since it was viewed as morally acceptable [57].

The current study found interparental violence, place of residence and region to be significantly related to IPV among PNG women. It was discovered that women who experienced interparental violence showed a greater likelihood of IPV experience. The study findings corroborate previous studies in Pakistan [58], Bangladesh [45], Nigeria [41], and Ethiopia [59] that being exposed to interparental violence increases women’s likelihood of IPV experience. As a result, the study results support previous studies and imply that this relationship exists in PNG as well. Prior research has proposed possible explanations for this association, including mechanisms by which being exposed to interparental violence may be linked to a greater danger of women experiencing IPV [41, 53, 60]. For example, it was suggested that women who have experienced interparental or intra-family abuse may develop psychological depictions of connections that make them more susceptible to IPV [40, 61]. Daughters might develop connection simulations along the dominance-subservience and victim-victimizer scopes due to seeing their father and mother strike one another [40, 62]. As a result, women can select spouses and surroundings that reflect their comprehension of what affairs are all about, who they are in affairs with, and what to anticipate from a spouse [40, 61]. Thus, the study findings are in line with the multi-generational impact of violence studies [63].

Another prospect is that women who have experienced interparental violence will perceive IPV as a regular aspect of intimate affairs, particularly in PNG, where intimate affairs are moulded and dictated by traditional concepts and conceptualizations. This confirms Kwagala et al. [64] study in Uganda stated that interparental violence exposure is an aspect of socialization, fostering views that approve IPV. As a result, domestic abuse could be an aspect of a lifelong cycle, starting with infant exposure to violence in the home and progressing into adulthood with violence in intimate affairs and homes [40, 61]. It is uncertain whether culprits of domestic abuse have an intergenerational or multigenerational influence. Regardless, the findings sturdily underscore the necessity for early detection of IPV and family intervention to lessen the possibility that abused mothers' children may suffer abuse as victims or culprits as adults.

In our study, place of residence was shown to be significantly associated with IPV among women in PNG. This could be related to cultural beliefs and customs including male dominance in decision-making, female inheritance, polygamy, and religious issues, which could make disclosing any IPV experience in rural PNG difficult [65]. It is also likely that there was under-reporting among rural women in PNG, which could be related to the sensitivity around gender-based abuse and discussing female issues in the PNG environment, including rejection, embarrassment, or stigma connected with domestic violence [52, 66]. As a result, more research is required to explain why there are discrepancies between rural and urban women in PNG. Furthermore, it is common knowledge that urban women are typically financially autonomous and educated and that such women are perceived as a danger by their husbands. IPV may be used as a mechanism by men to exert control over their female spouses [67]. It is also possible that some urban women in PNG approve of wife-beating as a result of their exposure to interparental violence and financial reliance on men [41, 47]. In their relationships, such women frequently become helpless and ostracized, demonstrating an inability to safely criticize their partners and avoid violence [59].

The present study also revealed women living in the Highlands region have a greater probability of experiencing IPV relative to women living in the Southern region. This might be a result of the region's high population density, the dominance of men and masculine values, as well as the submissive dependency of women on men for survival in this area. Men in the highlands of PNG are said to deliberately oppose any increase in women's power because they perceive it as a loss for themselves. When their authority over women is questioned, men will use the bride-price argument, which claims that paying the bride price gives them complete control over their wives [68]. Traditionally, wedlock in the highlands of PNG is frequently understood to involve the handover of conjugal rights, granting the husband access to and control over the wife's body sexually. When individuals talk about "purchasing a woman/wife," they are referring to the trade of bride price, which is progressively seen as a type of commodity exchange [68]. With this in mind, women are more likely to experience IPV than these men.

## Strengths and limitations

To the best of our knowledge, our study is the first to examine the association between exposure to interparental violence and the experience if IPV among women using a nationally representative dataset. Furthermore, the usage of a nationally representative survey (DHS) allowed for the collection of an extremely representative sample of the target population. Conclusions from the study findings are valid due to the high sample size and nationally representative nature of the data. Nevertheless, the study's conclusions had some limitations. To begin with, the study depended on cross-sectional data, which limits causal explanations of the results. Secondly, because the study depended on self-reported data that may not be objectively checked, the prevalence of IPV and interparental violence may be underestimated or overstated. Furthermore, the data for this study was restricted to women alone, which is comparable with the widespread perception that women are the most common sufferers of IPV. Whereas this widely held assumption may be challenged in the future, the findings of this present study provide timely and valuable insights that may be utilized to tackle the existing oppression of women in domestic violence in PNG.

### Policy and public health implications

The discovery that exposure to interparental abuse augments the risk of IPV has policy and public health consequences. The findings of the study suggest that prevailing policies and programs be consolidated, or that new strategies and programs be developed to tackle interparental violence and IPV in PNG. Due to the complexities of interparental violence and its links to IPV, coupled with demographic considerations, single strategies and programs are improbable to result in long-term change and consequences. As a result, multiple and comprehensive techniques and approaches are necessary. The supply of information and capital at the communal and societal levels, as well as emancipation programs for women, expanded social systems, and self-assurance for women, are all feasible measures for fighting interparental violence. Young women subjected to interparental violence must be shown special consideration in these intercession strategies. The majority of societies, especially in urban areas in PNG, need to increase public health edification and information about the serious health and communal repercussions of interparental violence and IPV. Because of concerns such as poverty, lack of access to proper domestic violence evidence and services, insufficient legitimate reparation for sufferers of abuse, and traditional customs, morals, and practices, policies for tackling interparental violence and IPV face particular challenges in PNG settings. Nevertheless, initiatives and interventions that are attentive to the cultural environment of people who are engaged in interparental violence and IPV may be the most effective in fostering long-term transformation and results.

## Conclusion

Exposure to interparental violence was found to be significantly associated with IPV among women in PNG. The findings of this study suggest the need for proven operational strategies to reduce IPV, such as improving anti-IPV laws in PNG. We recommend the development and implementation of intercession strategies to reduce the experience and justification of violence among women exposed to interparental violence. In addition, health professionals should implement counseling and health education initiatives to tackle the consequences of IPV on women’s well-being.

## Data Availability

Data for this study is available at https://dhsprogram.com/data/dataset/Papua-New-Guinea_Standard-DHS_2017.cfm?flag=1.

## Abbreviations

AIC: Akaike Information Criterion
aOR: Adjusted odds ratio
CI: Confidence Interval
DHS: Demographic and Health Survey
HIV: Human Immunodeficiency Virus
ICC: Inter-Cluster Correlation
ICF: Inner City Fund
IPV: Intimate Partner Violence
PNG: Papua New Guinea
STROBE: Strengthening Reporting of Observational Studies in Epidemiology
VIF: Variance Inflation Factor
